# Likelihood of admission to hospital from the emergency department is not universally associated with hospital bed occupancy at the time of admission

**DOI:** 10.1002/hpm.3086

**Published:** 2020-10-09

**Authors:** Ellen Tolestam Heyman, Martin Engström, Amir Baigi, Lina Dahlén Holmqvist, Markus Lingman

**Affiliations:** ^1^ Emergency Department Region Halland Varberg Sweden; ^2^ Department of Public Health and Community Medicine Institute of Medicine Sahlgrenska Academy University of Gothenburg Gothenburg Sweden; ^3^ Department of Healthcare, Region Halland Central Office Region Halland Sweden; ^4^ Department of Anaesthesia and Intensive Care, Medicine Lund University Lund Sweden; ^5^ Department of Research and Development Region Halland Halmstad Sweden; ^6^ Department of Molecular and Clinical Medicine Institute of Medicine Sahlgrenska Academy University of Gothenburg Gothenburg Sweden; ^7^ Emergency Department Sahlgrenska University Hospital Gothenburg Sweden; ^8^ Halland Hospital Group, Region Halland Sweden; ^9^ Department of Molecular and Clinical Medicine/Cardiology Institute of Medicine Sahlgrenska Academy University of Gothenburg Gothenburg Sweden

**Keywords:** admission rate, disposition decision, emergency department, emergency medicine, hospital bed occupancy

## Abstract

**Background:**

The decision to admit into the hospital from the emergency department (ED) is considered to be important and challenging. The aim was to assess whether previously published results suggesting an association between hospital bed occupancy and likelihood of hospital admission from the ED can be reproduced in a different study population.

**Methods:**

A retrospective cohort study of attendances at two Swedish EDs in 2015 was performed. Admission to hospital was assessed in relation to hospital bed occupancy together with other clinically relevant variables. Hospital bed occupancy was categorized and univariate and multivariate logistic regression were performed.

**Results:**

In total 89,503 patient attendances were included in the final analysis. Of those, 29.1% resulted in admission within 24 h. The mean hospital bed occupancy by the hour of the two hospitals was 87.1% (SD 7.6). In both the univariate and multivariate analysis, odds ratio for admission within 24 h from the ED did not decrease significantly with an increasing hospital bed occupancy.

**Conclusions:**

A negative association between admission to hospital and occupancy level, as reported elsewhere, was not replicated. This suggests that the previously shown association might not be universal but may vary across sites due to setting specific circumstances.

## 1 BACKGROUND

High hospital bed occupancy and its potential associated risks have been widely discussed in recent years.^1^ In The Organisation for Economic Co‐operation and Development countries, a reduction in the number of beds available for in‐patient care has been reported, not least in Sweden which has one of the lowest numbers of beds per capita.^2^ At the same time, a general increase in hospital bed occupancy is seen in most countries around the world.^2^ In many countries a reduction in hospital capacity is in line with policies to limit public spending on health.^2^ In parallel, an increasing number of patients are seeking care at Emergency Departments (EDs) internationally.^3^, ^4^, ^5^, ^6^ It is therefore argued that EDs play a major role in a general shift from inpatient to outpatient care.^4^ At the same time the admission decision has to be medically favourable and safe for the patient. Altogether, the decision whether to admit or discharge a patient from the ED is considered to be increasingly important and challenging.

Previous studies conclude that high hospital bed occupancy is associated with ED exit block, boarding of patients in the ED and a longer ED length of stay.^1^, ^7^, ^8^, ^9^, ^10^ Conversely, a sudden decrease in hospital bed occupancy is associated with a decreased ED occupancy and mean ED length of stay.^11^ Boarding, in turn, has been pointed out as one primary cause of ED overcrowding, that is when identified need for emergency services exceeds available resources in the ED.^12^, ^13^ Overcrowding is easy for ED staff to recognize but difficult to define and measure. According to a systematic review, 71 unique measures are currently in use.^14^

A few studies explore if there is an association between hospital bed occupancy and the likelihood of admission from the ED. In one survey by Blom et al. of all ED visits^15^ and another of ED visits with acute abdominal pain,^16^ the likelihood of admission decreases when hospital bed occupancy increases. Furthermore, in a study by af Ugglas et al. of all patients older than 17 years in six EDs,^17^ higher hospital occupancy is associated with a decline in admissions for inpatient care. We aimed to assess whether these previous results suggesting an association between hospital bed occupancy and likelihood of admission to hospital from the ED can be reproduced in a different study population.

## 2 METHODS

### 2.1 Study design

In this retrospective multicentre cohort study, all ED attendances within the Swedish region of Halland from the First of January to the 31st of December 2015 were assessed. It was regarded as an attendance when the patient was registered in the patient administration system upon arrival. Psychiatric emergencies were excluded since their care was given by separate wards with their own EDs. No further exclusion of patients was made.

The study was part of the project *Defining care processes and predicting risk for adverse events by applying machine learning algorithms to the Region Halland data warehouse*, approved by The Ethics Committee in Lund (Dnr 2016/517).

### 2.2 Setting

Two emergency hospitals serve the about 325,000 inhabitants living in the region of Halland, in the southwestern part of Sweden. During the time of the study, hospital A had 293 hospital beds on average and approximately 48,000 annual ED attendance. Hospital B had on average 219 hospital beds and received approximately 41,000 ED visits yearly.

In the ED at Hospital A, patients were assessed and treated by physicians on duty, mostly within the medical, surgical, orthopaedic or paediatric fields. At Hospital B, care was not only given by physicians on call but also, and more frequently, by emergency physicians.

### 2.3 Data collection

Upon arrival to either of the two EDs, patients were registered in the patient administrative system by a secretary. All patients were triaged by the Rapid Emergency Triage and Treatment System (RETTS), the most common triage system used in Swedish EDs.^18^ The RETTS algorithm prioritizes patients by vital parameters and main complaints into five triage categories, one to five, with category 1 being the highest level of urgency and five being the lowest. During the ED stay the triage level in some cases changed due to altered patient status. The triage level in this study refers to the last level recorded in the system.

Data from the Region Halland's data warehouse were used.^19^ Patient data were retrieved from Region Halland's electronic health record system Vård Administrativt System. All data generated through each patient visit were linked to a unique identifier. The dependent variable considered was ‘Emergency admission to one of the two hospitals within 24 h’. The 24 h started upon registration on arrival. Hospital bed occupancy was recorded by the hour and measured over all hospital wards according to hospital standard measurement and report of occupancy. It was defined as the number of inpatients by the hour and hospital and was divided by the total number of accessible hospital beds at the same date. Data from psychiatric wards and intensive care units were excluded from the hospital bed occupancy bed calculation. The decision to admit or not was linked to hospital bed occupancy measured by the same hour and at the same hospital as ‘departure’ was registered in the ED information system. ‘Departure’ marks the time when the patient is physically leaving the ED and entering the in‐hospital ward or being discharged and is used as a proxy for the time of the disposition decision.

### 2.4 Statistical analyses

Variables included in the model were identified by experienced clinicians and administrative staff, with the aim of including all observable variables with a possible impact on the decision to admit a patient from the ED. Hospital bed occupancy was categorized into five intervals to enable comparison between different levels of occupancy and identification of potential non‐linear associations. Descriptive statistics were used to highlight the nature and distribution of the variables. For dichotomous and category variables, percentage (%) was calculated and for continuous variables, both means and medians were determined. As a statistical dispersion, both standard deviation (SD) and interquartile range were assessed.

For analysing the relationship between emergency admission to the hospital within 24 h and the characteristic variables, univariate and multivariate logistic regression with odds ratio (OR) and confidence interval was performed. Association between emergency admission to hospital and the following variables was explored: Hospital bed occupancy, sex, age, which hospital, referral or not, arrival by ambulance or not, time of arrival to the ED, day of the week and emergency triage priority level. The Student's *t*‐test was performed for comparisons between groups. *p*‐value was calculated and the significance level in the study was set to 0.05.

The distribution of bed occupancy differed between the hospitals. Therefore, a sensitivity analysis was performed with separate analyses for each hospital to clarify that the hospital's covariates didn't counteract each other and affected the result.

The statistical software used for the statistical analysis was SPSS version 26.0.^20^

## 3 RESULTS

### 3.1 Descriptive statistics

There were 89,670 registered attendances to the two EDs during the one‐year study of which 167 patients (0.2%) were excluded due to incompleteness of data. The remaining 89,503 patients were included in the analysis. Among the patients attending the EDs during this time, 29.1% were admitted to one of the two hospitals within 24 h. The mean hospital bed occupancy of the two hospitals during the study period, recorded by the hour and measured over all hospital wards besides psychiatric wards and intensive care units, was 87.1% (SD 7.6; Table 1).

**TABLE 1:** Descriptive statistics on the study variables of the ED visits included in the study (*N* = 89,670)

		Bed occupancy					
		All visits	<85%	85%–89%	90%–94%	95%–98%	≥99%
ED visits. No. (%)		89,670 (100)	34,825 (38.8)	22,099 (24.6)	18,972 (21.2)	9215 (10.3)	4559 (5.1)
Hospital bed occupancy level	Mean (SD)	87.05 (7.6)	79.42 (4.2)	87.52 (1.4)	92.32 (1.4)	96.77 (1.2)	101.52 (2.0)
	Median (IQR)	87.24 (81.8–92.5)	80.27 (77.0–82.8)	87.58 (86.3–88.8)	92.21 (91.1–93.5)	96.71 (95.7–97.7)	101.03 (100.0–102.6)
	Min‐max	60.84–111.11					
Admission from ED within 24 h^a^. No. (%)	No admission	63,534 (70.9)	25,079 (72.0)	15,553 (70.4)	13,210 (69.6)	6491 (70.4)	3201 (70.2)
	Admission	26,136 (29.1)	9746 (28.0)	6546 (29.6)	5762 (30.4)	2724 (29.6)	1358 (29.8)
Sex. No. (%)	Man	45,871 (51.2)	17,821 (51.2)	11,251 (50.9)	9728 (51.3)	4728 (51.3)	2343 (51.4)
	Woman	43,799 (48.8)	17,004 (48.8)	10,848 (49.1)	9244 (48.7)	4487 (48.7)	2216 (48.6)
Age (years)	Mean (SD)	46.25 (28.5)	44.74 (28.9)	46.43 (28.7)	47.35 (28.3)	48.00 (27.7)	48.88 (26.9)
	Median (IQR)	48.00 (21.0–71.0)	46.00 (20.0–71.0)	49.00 (21.0–72.0)	50.00 (22.0–72.0)	50.00 (23.0–72.0)	52.00 (25.0–72.0)
	Min‐max	0–105					
	Missing	20					
Hospital. No. (%)	Hospital A	48,207 (53.8)	26,626 (76.5)	11,346 (51.3)	7062 (37.2)	2421 (26.3)	752 (16.5)
	Hospital B	41,463 (46.2)	8199 (23.5)	10,753 (48.7)	11,910 (62.8)	6794 (73.7)	3807 (83.5)
Referral from other care giver. No. (%)	No	77,975 (87.0)	30,236 (86.8)	18,891 (85.5)	16,444 (86.7)	8234 (89.4)	4170 (91.5)
	Yes	11,695 (13.0)	4589 (13.2)	3208 (14.5)	2528 (13.3)	981 (10.6)	389 (8.5)
Mode of arrival. No. (%)	Walk‐in	65,623 (73.2)	25,790 (74.1)	16,130 (73.0)	13,729 (72.4)	6667 (72.3)	3307 (72.5)
	Ambulance	24,047 (26.8)	9035 (25.9)	5969 (27.0)	5243 (27.6)	2548 (27.7)	1252 (27.5)
Time of arrival (h)	00–06	9523 (10.6)	2578 (7.4)	1983 (9.0)	2265 (11.9)	1634 (17.7)	1063 (23.3)
	06–12	26,512 (29.6)	9254 (26.6)	6582 (29.8)	5474 (28.9)	3278 (35.6)	1924 (42.2)
	12–18	32,995 (36.8)	15,670 (45.0)	8598 (38.9)	6232 (32.8)	1903 (20.7)	592 (13.0)
	18–24	20,640 (23.0)	7323 (21.0)	4936 (22.3)	5001 (26.4)	2400 (26.0)	980 (21.5)
Weekday. No. (%)	Mon–Thur	51,759 (57.7)	13,143 (37.7)	14,432 (65.3)	13,809 (72.8)	7033 (76.3)	3342 (73.3)
	Fri	12,315 (13.7)	8513 (24.4)	1813 (8.2)	1201 (6.3)	465 (5.0)	323 (7.1)
	Sat–Sun	25,596 (28.5)	13,169 (37.8)	5854 (26.5)	3962 (20.9)	1717 (18.6)	894 (19.6)
Emergency triage category. No. (%)	1	3312 (3.7)	1216 (3.5)	794 (3.6)	735 (3.9)	375 (4.1)	192 (4.2)
	2	19,545 (21.8)	7759 (22.3)	4920 (22.3)	4046 (21.3)	1930 (20.9)	890 (19.5)
	3	42,840 (47.8)	16,118 (46.3)	10,616 (48.0)	9375 (49.4)	4469 (48.5)	2262 (49.6)
	4	10,056 (11.2)	4351 (12.5)	2452 (11.1)	1844 (9.7)	952 (10.3)	457 (10.0)
	5	13,770 (15.4)	5352 (15.4)	3283 (14.9)	2931 (15.4)	1465 (15.9)	739 (16.2)
	Missing	147 (0.2)					

Abbreviations: ED, emergency department; IQR, interquartile range; SD, standard deviation.

^a^ Admission from ED to any of the two hospitals within 24 h after registration on arrival.

### 3.2 Association between hospital admission and the clinical variables

In neither the univariate nor the multivariate analysis with reference category set to occupancy level of <85%, an increased hospital bed occupancy level was associated with a decreased likelihood of admission to the hospital from the ED (Table 2).

**TABLE 2:** Association between Admission from ED within 24 h^a^ and hospital bed occupancy and well as other clinical variables

		Univariate			Multivariate		
		OR	95% CI	*p*‐value	OR	95% CI	*p*‐value
Hospital bed occupancy level (categorized variable) (%)	<85% (ref)			<0.001			0.102
	85%–89%	1.08	1.04–1.12	<0.001	1.04	0.99–1.09	0.088
	90%–94%	1.12	1.08–1.17	<0.001	1.07	1.02–1.12	0.011
	95%–98%	1.08	1.03–1.14	0.003	1.01	0.95–1.08	0.735
	≥99%	1.09	1.02–1.17	0.011	1.05	0.96–1.14	0.274
Sex	Man (ref)						
	Woman	0.98	0.95–1.01	0.167	0.95	0.92–0.99	0.005
Age (years)		1.03	1.03–1.03	<0.001	1.02	1.02–1.02	<0.001
Hospital	Hospital A (ref)						
	Hospital B	1.05	1.02–1.08	0.002	0.96	0.92–0.99	0.018
Referral from other care giver	No (ref)						
	Yes	1.60	1.54–1.67	<0.001	1.79	1.71–1.88	<0.001
Mode of arrival	Walk‐in (ref)						
	Ambulance	5.85	5.67–6.04	<0.001	2.77	2.67–2.88	<0.001
Time of arrival (hrs)	00–06 (ref)			<0.001			<0.001
	06–12	0.85	0.81–0.90	<0.001	0.76	0.72–0.81	<0.001
	12–18	0.81	0.77–0.85	<0.001	0.77	0.73–0.82	<0.001
	18–24	0.71	0.67–0.75	<0.001	0.88	0.83–0.94	<0.001
Weekday	Mon–Thur (ref)						<0.001
	Fri	0.99	0.95–1.03	0.521	1.03	0.97–1.08	0.358
	Sat–Sun	0.80	0.78–0.83	<0.001	0.87	0.83–0.91	<0.001
Emergency triage category	1	18.17	16.47–20.04	<0.001	12.59	11.31–14.01	<0.001
	2	5.85	5.51–6.21	<0.001	4.35	4.08–4.64	<0.001
	3	1.53	1.45–1.62	<0.001	1.37	1.28–1.45	<0.001
	4 (ref)			<0.001			<0.001
	5	0.40	0.37–0.43	<0.001	0.55	0.51–0.60	<0.001

*Note:* Univariate and multivariate logistic regression was performed with hospital bed occupancy as a categorized variable. Goodness of fit as determinant (Nagelkerke *R*‐square) and test for collinearity (VIF) have been conducted^b^ (*N* = 89,503).

Abbreviations: CI, confidence interval; ED, emergency department; OR, odds ratio; VIF, variance inflation factor.

^a^ Admission from ED within 24 h (No admission = ref).

^b^ For all variables VIF <2. Nagelkerke *R*‐square = 0.36.

In all, the most predictive variable for emergency admission according to the multivariate analysis was emergency triage level 1 which was almost three times as strong as the second strongest predictor emergency triage level 2 followed by the third; mode of arrival.

Referral from other health care providers and visits on weekdays were associated with a higher likelihood of admission compared to non‐referred patients and visits on weekends respectively. The odds for admission were not equal between sites. Older patients were more likely to be admitted and men were slightly more likely to be admitted than women.

In the sensitivity analysis, separate analyses were performed for each hospital in relation to the occupancy level. The result was broadly equivalent for the three closest categories of occupancy level with the first category as a reference. However, the category with the highest occupancy level did not reach the significance level. OR for the significant results varied between (1.07–1.11; *p* < 0.05) for Hospital A and OR (1.09–1.12; *p* < 0.05) for Hospital B.

## 4 DISCUSSION

In this population wide study with almost complete coverage of patients presenting at the EDs of two acute care hospitals in Sweden, no negative association between hospital bed occupancy and likelihood of admission to the hospital was identified.

Our findings contrast previous reports, and suggests that a negative association between the likelihood of hospital admission and hospital bed occupancy is plausible, but might not be universal.

We expected the bed occupancy to be negatively associated with the ED disposition decision based on clinical knowledge about the work streams in the EDs included in the present report and based on two previous well conducted Swedish ED studies by Blom et al.^15^, ^16^ in which an unselected ED population and patients with abdominal pain are included and one study by af Ugglas et al.^17^ of six EDs in which adult patients are included. These studies with patients presenting at Swedish EDs find a lower likelihood of being admitted to inpatient wards during hospital crowding. One interpretation of Blom et al.'s studies is that some patients are discharged inappropriately from the ED when hospital bed occupancy is high. Another tentative explanation is that patients might be unnecessarily admitted when hospital bed occupancy is low. Our findings do not support either interpretation in the present setting.

Our result might be influenced by the mean hospital bed occupancy level during the study period. At the investigated hospital in Blom's studies, the mean hospital bed occupancy during the study period, measured hospital‐wide and by the hour of patient presentation, is 94.9%^15^, ^16^, ^21^ and in af Ugglas' study, measured hourly over all in‐hospital wards that care for adult emergency cases at six hospitals, is 93.3%^17^ compared to mean bed occupancy of 87.1% measured over all wards besides the psychiatric wards and intensive care units at the two hospitals in this study. Given the comparably lower average hospital bed occupancy observed at the present study sites, it might well be the case that access block is rare enough for the displacement effect previously described by Blom and af Ugglas to not have taken effect. It was traditionally stated that the risk of bed crisis emerges when the average hospital bed occupancy exceeds about 85% and that regular bed shortages and periodic bed crises can be expected if average hospital bed occupancy surpasses 90% in a theoretical model.^22^ But according to a survey among 23 Australian acute hospitals in 2012,^23^ modern hospital systems seem to be more robust and operate optimally up to higher levels in the 90th centile. Our study could support that claim. However, it has to be taken into consideration that Australian EDs work in a different context.

A contributory matter of our lack of negative association in this study might possibly be a relatively small hospital size. The average number of beds of the two hospitals in this study was 293 and 219 compared to 420 beds at the hospital in Blom's studies.^15^, ^16^ In the Australian study, critical levels of bed occupancy are observed at higher occupancy levels at smaller hospitals.^23^ Deterioration in ED performance, here succinctly defined as an increasing ED length of stay for admitted patients but not for discharged patients, is seen at an occupancy level of 98% in smaller hospitals with less than 300 beds compared to 86% in larger hospitals with more than 900 beds.^23^ According to this research, smaller hospitals seem to respond better to rising occupancy. The Australian authors therefore point out the need to calculate an optimal occupancy level for each hospital.

There might be other factors influencing our lack of negative association between likelihood of admission and hospital bed occupancy. One might be that physicians do not have an up‐to‐date real time overview of the hospital bed situation at the time of admission decision thus decoupling it from the disposition decision.

In the present report we confirm previous observations that admission rates are lower at week‐ends. This might be attributed to a greater degree of severity of illness among admitted patients during the weekends or to a slightly different casemix in weekends compared to weekdays with for instance a reduction in community referrals from family doctors.^24^, ^26^

A general mismatch in research on patient flow and crowding is seen; causes of ED overcrowding are generally found outside of the ED, causing input and output issues, whilst solutions are mostly searched for within the ED.^13^ Therefore, we do believe that this study, with hospital bed occupancy as an exposure contributes to a better understanding of the ED working process with it's system‐wide approach.

### 4.1 Limitations

Hospital bed occupancy level, our predictor of interest, was measured by the number of inpatients by the hour and hospital and was divided by the total number of accessible hospital beds at the same date. Data from the psychiatric wards and intensive care units were excluded. The denominator was not available by the hour hence our analysis relies on the assumption of that the total number of beds in the hospital is equally distributed throughout the day. While this assumption is fairly solid, we argue that it would not obscure a clear signal in the data.

The point of time for the admission decision was connected to hospital bed occupancy by the same hour as ‘departure’ was registered in the ED information system. ‘Departure’ marks the time when the patient is physically leaving the ED and entering the in‐hospital ward or being discharged. This proxy differs somewhat from the time of the actual admission decision but was the closest data available. Our clinical experience from the hospitals studied is that the difference between time of disposition decision and our proxy ED departure is short in comparison to changes in hospital bed occupancy levels.

Also, hospital bed occupancy was measured hospital‐wide rather than for specific departments. This could be misleading when the admission decision is dependent on available beds at specific wards. On the other hand, in the hospitals included in this report patients are usually placed in hospital wards that sort under departments other than that formally responsible for the patient when the hospital reaches full capacity. Therefore, we do believe that measuring hospital‐wide bed occupancy offers a more correct view given local routines. Importantly transfer of patients between hospitals for capacity is negligible in our setting.

When we lump the observations together and adjust for the hospital there is a possibility that the covariate dynamics is very different between the hospitals. The result from the sensitivity analysis was largely equivalent with the main analysis in this article. This should be a credit for the study's sensitivity to this issue.

Furthermore, emergency admission to hospital was recorded as admission within 24 h, starting upon registration on arrival. If the ED length of stay exceeded 24 h and the patient then would have been admitted, it would have been recorded as a discharge. However, this event is considered very uncommon in these EDs. In a comparison of connecting ED visits to admission within 24 h with admission within 48 h, the difference was only a handful patients throughout a year.

Finally, one has to be cautious when generalizing results concerning organizational issues since they might vary considerably between hospitals and countries. However, the issue of hospital bed shortages and ED overcrowding has been reported from all over the world.^26^

### 4.2 Implications

Further studies are needed in order to better understand how average level and variation in hospital bed occupancy affect the likelihood of admission and ED crowding in different settings. Specifically, potentially different patterns between high and low volume sites. We also see additional value in research aiming to better understand drivers and modifiers of patient flow through the ED.

## 5 CONCLUSIONS

In this population‐wide study, a negative association between the likelihood of admission to hospital and hospital bed occupancy level, as reported elsewhere, was not replicated. This suggests that the previously shown association might not be universal but may vary across sites due to differences such as average hospital bed occupancy level.

## AUTHOR CONTRIBUTIONS

Ellen Tolestam Heyman authored the article. Martin Engström supervized study design and interpretation of results. Amir Baigi performed statistical analysis. Lina Dahlén Holmqvist supervized interpretation of results and article writing. Markus Lingman supervized study design, interpretation of results and writing of the article. All authors read and approved the final manuscript.

## ETHICS STATEMENT

The study was part of the project Defining care processes and predicting risk for adverse events by applying machine learning algorithms to the Region Halland data warehouse, approved by The Ethics Committee in Lund (Dnr 2016/517). Participants were offered in local media to opt out but none of the included participants did. The study is a retrospective‐register study; the participants are not undergoing any intervention.
